# Temporal spectral evolution of pre-stimulus brain activity in visual and visuomotor tasks

**DOI:** 10.1007/s11571-022-09910-2

**Published:** 2022-11-30

**Authors:** Esteban Sarrias-Arrabal, Marika Berchicci, Valentina Bianco, Manuel Vázquez-Marrufo, Rinaldo Livio Perri, Francesco Di Russo

**Affiliations:** 1 Department of Experimental Psychology, Faculty of Psychology, University of Seville, Seville, Spain; 2 Department of Movement, Human and Health Sciences, University of Rome “Foro Italico”, Rome, Italy; 3 Santa Lucia Foundation (IRCCS Fondazione Santa Lucia), Rome, Italy; 4 University “Niccolò Cusano”, Rome, Italy

**Keywords:** Temporal spectral evolution (TSE), Event-related potential (ERP), Frequencies, Visual task, Pre-stimulus

## Abstract

The aim of this study was to describe the spectral features of pre-stimulus event-related potential (ERP) components elicited in visual tasks such as the Bereitschaftspotential (BP), prefrontal negativity (pN) and visual negativity (vN). ERPs are considered time-locked and phase-locked (evoked) activity, but we have also analyzed the non-phase but time-locked (induced) activity in the same interval by applying the temporal spectral evolution (TSE) method. Participants (N = 26) were tested in a passive task, a simple response task (SRT) and a discriminative response task (DRT), where EEG activity was recorded with 64 scalp electrodes. We analyzed the time-frequency modulations (phase and non-phase) prior to the onset of the stimuli in the sub-delta, delta, theta, alpha, beta, and gamma frequency bands. The results showed that all the pre-stimulus ERP components were mainly regulated by evoked activity in the sub-delta band. On the other hand, induced activity seems to be linked to evoked responses but with a different psychophysiological role. We concluded that other preparatory cognitive mechanisms associated with ERPs can also be detected by the TSE method. This finding may suggest underlying mechanisms in non-phase activity and requires the addition of non-phase activity analysis to the traditional analysis (phase and evoked activity).

## Introduction

The anticipatory activity represents a fundamental brain function, enabling humans to predict upcoming events and to prepare accordingly. In laboratory tasks requiring stimulus processing and a motor response, electrophysiological recordings allow us to investigate anticipatory brain activity in response to stimulus presentation and associated motor responses. Using event-related potential (ERP) measures, several slow cortical potentials reflecting anticipatory activity have been reported; in particular, contingent negative variation (CNV) (Walter et al. 1964) emerges during the interval between the presentation of a cue (S1) and the imperative stimulus (S2) and stimulus-preceding negativity (SPN) recorded before the stimuli conveying rewards about past performance, instructions about future tasks, or anticipating an affective stimulus (e.g., Gevins et al. 1989; for a review see Van Botxel and Böcker 2004). Other studies have investigated the fore period of stimuli signaling the upcoming task (e.g., Blangero and Kelly 2017; Wolff et al. 2018), intentional task switching (Astle et al. 2012) or visually guided (MacKay et al. 1986) saccades. Often, in these paradigms, the presence of a cue does not allow the isolation of purely endogenous cortical activity because it overlaps with exogenous activities evoked by cue onset (Di Russo et al. 2020).

In paradigms without a cue, a family of preparatory ERPs has been identified. In particular, three components have been described: (1) The Bereitschaftspotential (BP) or readiness potential (RP) arising from premotor areas and areas associated with motor preparation for voluntary movements (e.g., Kornhuber and Deecke 1965; Shibasaki and Hallett 2006), including externally triggered movements (e.g., Cunnington et al. 2002; Di Russo et al. 2017). Many studies have shown that late CNV corresponds to BP (e.g., Donchin et al. 1972; Gaillard 1977; Grünewald et al. 1979; Rohrbaugh and Gaillard 1983); (2) prefrontal negativity (pN) arising from the prefrontal cortex (PFC) and areas associated with cognitive (mainly inhibitory) preparation in discriminative visual tasks (e.g., Berchicci et al. 2012; Bianco et al. 2017); and (3) visual negativity (vN) originating from the extrastriate visual cortex and areas associated with sensory preparation (Bianco et al. 2019a, b; Di Russo et al. 2019) in both passive vision tasks and visuomotor tasks requiring simple and discriminative motor responses. While several studies have investigated the BP component, a limited number of studies have investigated the pN and the vN (for a review on the BP and the pN, see Di Russo et al. (2017); for normative data on the BP, the pN and the vN, see Di Russo et al. (2019)). Relevant to the present study, to the best of our knowledge, the spatiotemporal evolution of the pN and vN components has only been studied using ERP analysis, and the spectral features of these components are unknown.

Regarding pre-stimulus evoked (phase) activity of ERPs, Barry et al. (2010, 2014), using the fast Fourier transform (FFT), observed that prior to the presentation of the imperative stimulus, both delta and alpha bands contribute to the CNV wave in the same interval. An EEG study using self-paced movements found that during movement preparation, alpha and beta band activities are both present in the supplementary motor area (Ohara et al. 2000), presumably generating BP. Moreover, Kim et al. (2017), through the application of a self-paced hand grasping task, also suggested that beta contributes to the generation of BP. In fact, Shibasaki and Hallett (2006 reported an increase in desynchronization in the beta band in central regions before movement onset. However, the EEG signal is composed not only of evoked (time-locked and phase-locked) but also induced (time-locked and non-phase-locked) activity (Cohen 2014). The latter activity can be obtained by applying temporal spectral evolution (TSE) analysis (originally described by Salmelin and Hari 1994). With TSE, we can extract crucial time-frequency information non-phase-locked (induced) activity with certain events from the EEG traces (see Agyei et al. (2015, 2016); Lehtela et al. (1997); Ohara et al. (2000); Van der Meer et al. (2008; Van der Meer and van der Weel (2017); and Vilhemsen et al. (2018) for technical characteristics of the TSE method). According to Hari et al. (1997), the advantage of the TSE over other power analysis methods is that information obtained by amplitude analysis shares common features with typical ERP analysis in the time domain.

The TSE method has been commonly used to study the induced (non-phase) spectral features in the same time interval of ERP components following the onset of a stimulus (cue or target), including the P1, N1, P2, N2, and P3 components in the visual domain (e.g., Rihs et al. 2007; Sannita et al. 2001; Vázquez-Marrufo et al. 2001a, b;zquez-Marrufo et al. 2019). TSE can be extracted from EEG signal activity that is not in phase with the stimuli but that is modulated by the demands of the task. Since TSE represents non-phase activity with stimuli, this opens a wide field of study and knowledge because it allows us to describe cognitive, motor and/or sensory processes that are not represented in ERPs. It is true that using techniques such as the FFT, it is possible to observe increases or decreases in the power of the spectral activity, but it is not possible to discern whether the observed increase or decrease occurs in phase or non-phase activity. In fact, previous studies have shown that non-phase alpha activity may represent a decrease in neural noise to allow other processes operating in other bands to be executed (Sarrias-Arrabal et al. 2021; Vázquez-Marrufo et al. 2019, 2020a). Nevertheless, induced pre-stimulus (non-phase) activity has hardly been analyzed by TSE.

Based on the above and with reference to the Di Russo group’s ERP studies, the first aim of the present study was to confirm with both ERP and TSE methods that the vN is present in all three tasks: the BP in the simple response task (SRT) and discriminative response task (DRT) and the pN in the DRT only. The second aim was to confirm the scalp topography of the spectral components that should mirror the distributions observed in ERP studies, i.e., bilateral parieto-occipital for the vN, medial centro-parietal for the BP, and prefrontal for the pN. Finally, our third aim was to analyze the induced activity associated with the subtended anticipatory processes and disentangle this activity from evoked activity.

## Methods

### Participants

Twenty-six participants (12 females) were recruited for this study. The mean age was 26.3 ± 1.5 years (range 18–40 years). The inclusion and exclusion criteria of individuals were the following: healthy adults (no history of neurological, psychiatric, or chronic somatic disease); high-quality EEG signal, that is, an activity not contaminated by considerable electrooculographic (EOG) and electromyographic (EMG) artifacts defined as an amplitude not exceeding ± 80 µV for more than 20% of recording time; right-handedness (Edinburgh handedness inventory, Oldfield 1971); normal or corrected-to-normal vision; absence of reported psychoactive or vasoactive medication. The participant’s written informed consent was obtained according to the Declaration of Helsinki after approval by the ethical committee of the IRCCS Santa Lucia Foundation.

### Stimuli and task design

**Fig. 1:**
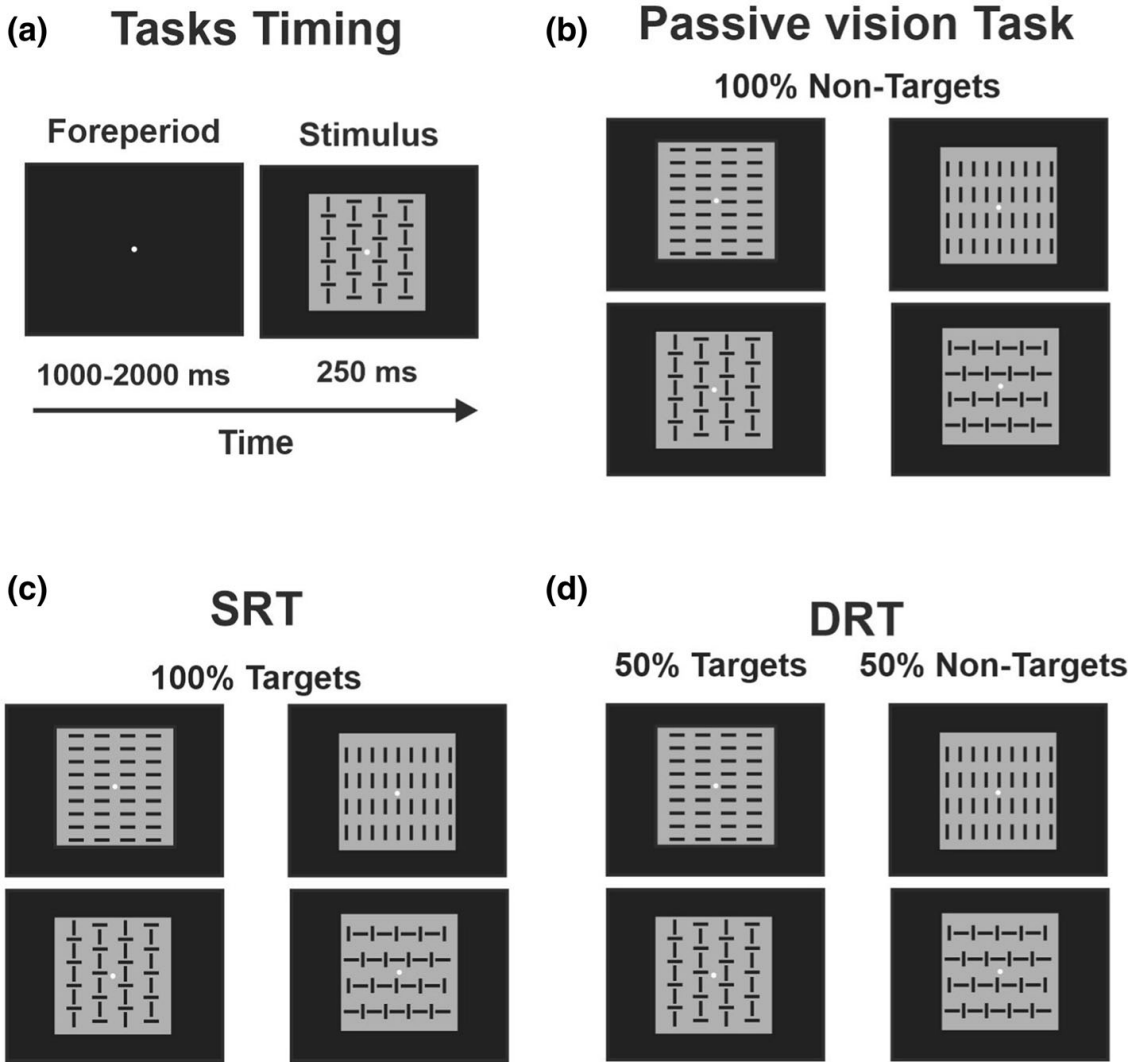
Schematic representation of the three tasks performed by the participants. **a** Timing common to all tasks. **b** In the passive vision task all stimuli do not require responses. **c** In the simple response task (SRT) all stimuli require a motor response. **d** In the discriminative response task (DRT) half of the stimuli do require a motor response, and the other half do not require responses

After the EEG cap was mounted on the scalp, each participant was tested in a sound-attenuated dimly lit room. Participants were comfortably seated 114 cm in front of a 24” computer screen with a response button pad positioned under their right index finger. In the center of the screen, a fixation point was present (0.15° diameter circle) that never disappeared to ensure attention on the task. Four visual stimuli (i.e., squared configurations extending 4 × 4° and vertical and/or horizontal bars) were randomly displayed for 250 ms with equal probability (p = 0.25); the stimulus-onset asynchrony varied from 1 to 2 s to prevent stimulus prediction and ERP overlaps with the previous and following stimuli. All participants performed three tasks: (1) a passive vision task in which they did not need to respond, but only observe stimuli displayed on the screen; (2) a visuomotor simple response task (SRT), in which they had to respond as soon as possible to any presented stimulus; (3) a discriminative response task (DRT), in which two stimuli were defined as targets (p = 0.5) and two as non-targets (p = 0.5). Participants were asked to be very accurate in discriminating the stimuli and to respond as soon as possible when the target was displayed on the screen, withholding the response when a non-target was displayed. The presentation order of the four stimuli was randomized. The number of trials for any task (passive, SRT, DRT target, and DRT non-target) ranged from 340 to 440. These were presented in runs of approximately 2.5 min, followed by a brief rest period. Each task duration was approximately 20 min, depending on the individual rest time between runs. The order of the tasks was counterbalanced across participants. A representation of the stimuli and tasks is shown in Fig. 1.

### EEG data recording and analysis

The EEG was recorded using two standard BrainAmp™ amplifiers (BrainProducts, Germany) with 64 active electrodes (Acticap™, BrainProducts, Germany). In addition, a third BrainAmp™ amplifier (ExG type) was used to record the electrooculogram (EOG) with four electrodes configured in two bipolar pairs: one pair of electrodes placed on the left and right canthi (HEOG), and the second pair placed below and above the left eye (VEOG). This montage allowed us to record 64 EEG scalp channels and 4 EOG channels. Ag/AgCl sintered electrodes were used. EEG channels were placed according to the 10–10 International system, initially referenced to M1, and then offline re-referenced to the M1–M2 average. The EEG was digitized at 250 Hz, amplified (bandpass of 0.01–60 Hz including a 50 Hz notch filter), and stored for offline averaging. Data were recorded using Recorder 1.21 software and analyzed using Analyzer 2.2 software (BrainProducts, Germany). Removal of ocular artifacts was performed using independent component analysis (ICA) on the raw EEG signals (e.g., Jung et al. 2000). The EEG signals were segmented into epochs from 1100 ms prior to the stimulus to 900 ms after stimulus onset. A baseline correction (− 1100 to − 900 ms) was applied to all tasks. Artifact rejection was conducted to reject epochs still affected by amplitudes exceeding ± 50 µV. Approximately 8% of trials were rejected.

**Fig. 2:**
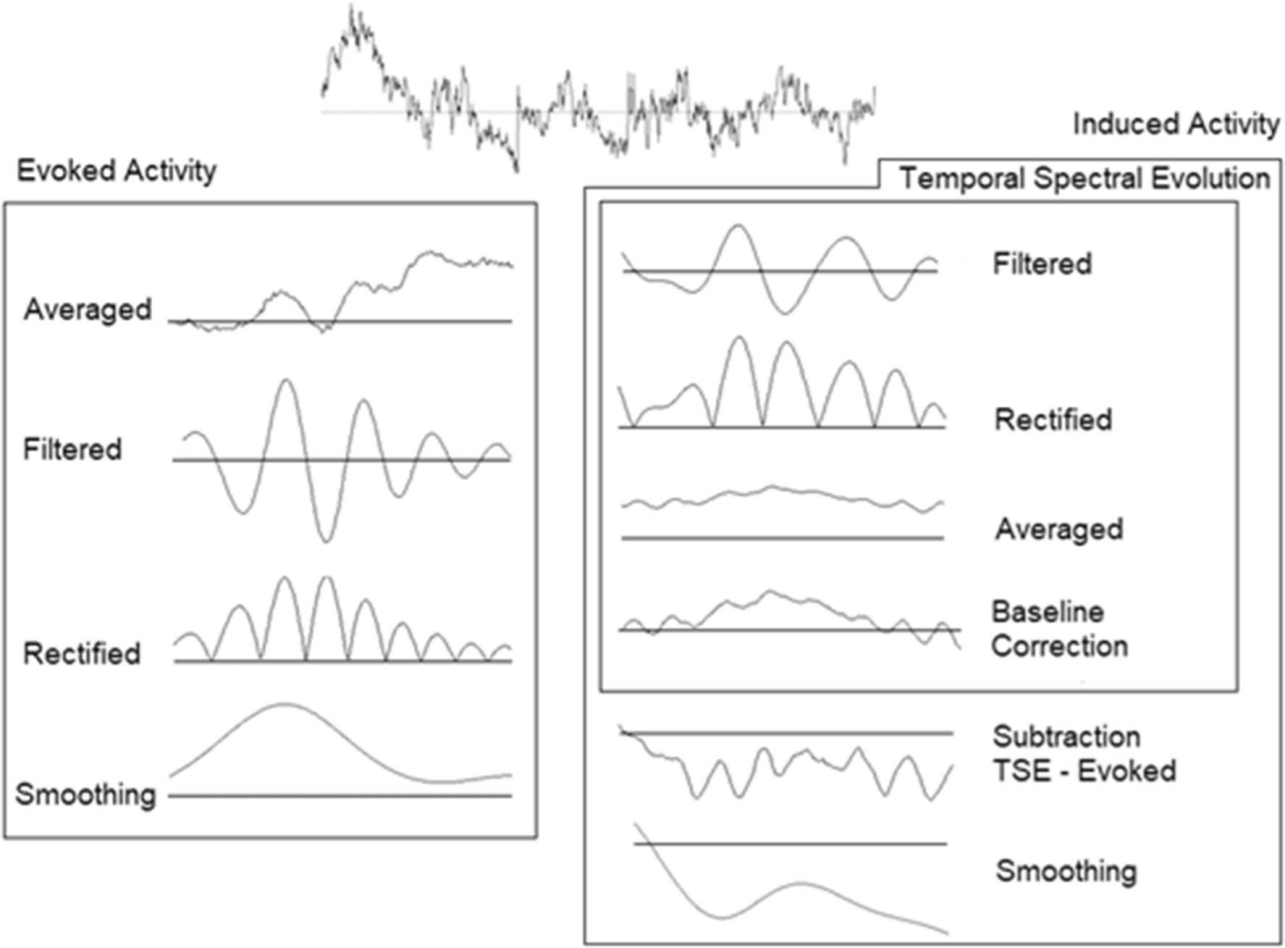
Representation of the temporal-spectral evolution (TSE) methodology

Both ERP and time-frequency analyses (TSE) were performed. For ERPs, the trials were simply averaged. For spectral evoked TSE analyses, the average ERP was bandpass filtered to achieve evoked activity in the following frequency bands: subdelta 0.5–2 Hz; delta 2–4 Hz; theta 4–8 Hz; alpha 8–13 Hz; beta 13–30 Hz; gamma 30–45 Hz. The filter slope was 12 dB/oct. The signal was then rectified (instead of squared) to obtain positive values without modifying the signal amplitude. As previously done, for display purposes, a 5 Hz low-pass filter (12 dB/oct slope) was used to smooth the signal (Enatsu et al. 2014; Lasaponara et al. 2019). To obtain induced responses, the mentioned bandpass filters were applied to the segmented EEG, the signal was rectified, and trials were averaged. A subtraction of evoked activity from TSE was subsequently performed to calculate the induced activity (Hari et al. 1997, Vazquez-Marrufo et al. 2020a; Sarrias-Arrabal et al. 2021) (Fig. 2).

### Statistical analysis

The collapsed localizers approach was used (e.g., Luck and Gaspelin 2017) to select the region of interest (ROI) and the time window for the statistical analyses. Following this method, all tasks and conditions were averaged, and the resulting waveforms were inspected for (1) global field power (GPF), (2) scalp mapping, and (3) normative data on the present tasks (Di Russo et al. 2019). Briefly, we selected the pre-stimulus interval where the GPF reached 80% of the maximum (which was time 0). In this interval, we selected frontal central and parieto-occipital electrodes where the amplitude was 80% of the maximum. Based on these analyses, the relevant electrodes were pooled to obtain three regions of interest (ROIs): the prefrontal ROI (AF7, AF3, Fp1, Fp2, AF4, AF8) indexing the pN component, the central ROI (FCz, Cz, CPz) indexing the BP component and the parieto-occipital ROI (PO7, PO3, O1, O2, PO4, PO8) indexing the vN component. Following these calculations, the mean activity of the time window from − 450 to 0 ms was considered and exported for statistical analysis of each ROI (prefrontal, central and parietal-occipital), task (passive, SRT and DRT), EEG analysis (ERP, evoked TSE, induced TSE), and TSE frequency band (subdelta, delta, theta, alpha, beta and gamma).

Preliminary analyses tested the ERP waveforms in the selected intervals and ROIs against zero (Student’s t-test) for all three tasks and for all ERP components. These analyses were performed to determine whether the studied activity was significantly different from the neural noise, which would consequently indicate reliable preparatory activity. Based on this analysis, ERP amplitude was analyzed by repeated measure analysis of variance (ANOVA) with the following factors: task (DRT, SRT, and passive) and ROIs (prefrontal, central and parietal-occipital). The same procedure was applied to TSE data in each frequency band.

Correlation analyses were performed using the Pearson r coefficient to study the relationship between the ERP and TSE amplitude to further evaluate and quantify which frequency band correlates with ERP at the amplitude level. Correlations were run for each ROI and task between ERP and any frequency band of TSE.

All statistical analyses were performed using Statistica 13.1 software. Mauchly’s test of sphericity was used to test the ANOVA prerequisites, and non-significant results (p > 0.05) for all considered comparisons indicated that the assumption of sphericity was not violated. For post hoc comparisons, the conservative Bonferroni test was used, which indicates adjusted p-values that are divided by the number of comparisons. To evaluate the effect size, the partial eta squared (pη^2^) was reported. The alpha level was set at 0.05.

## Results

**Fig. 3:**
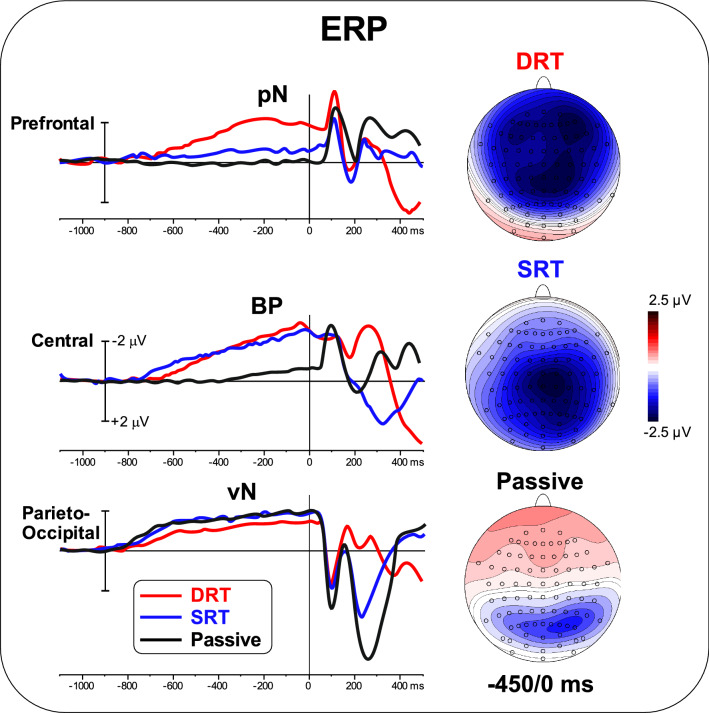
The left panel shows the overlapping ERP waveforms for the three ROIs during the three tasks. The right panel shows the scalp topography (top-flat view) for the three tasks in the − 450/0 ms window

Figure 3 shows the ERP waveforms for the three ROIs during the three tasks. The scalp topography in the − 450 to 0 ms epoch used for statistical analysis is also shown. Qualitatively, the pN, with a bilateral prefrontal distribution, was clearly present in the DRT, less present in the SRT, and absent in the passive task. BP, with a medial centroparietal distribution, was present in the DRT and the SRT but absent in the passive task. The vN, with a bilateral parieto-occipital distribution, was equally present in all tasks. ANOVA on the prefrontal ROI (the pN) was significant [F_(2,50)_ = 15.02; p < 0.0001; pη^2^ = 0.375]. Post hoc comparisons showed that pN was larger in the DRT (p < 0.0001) than in the other two tasks and that the SRT amplitude was larger (p < 0.01) than in the passive task. ANOVA on the central ROI (BP) was significant [F_(2,50)_ = 9.54; p < 0.005; pη^2^ = 0.276]. Post hoc comparisons showed that the BP in the passive task was smaller (p < 0.005) than that in the other two tasks, i.e., SRT and DRT, which did not differ from each other in the two tasks. ANOVA on the parieto-occipital ROI (the vN) was not significant [F < 1]. Table 1 shows the mean amplitude of the pre-stimulus ERP components.

**Table 1:** Mean amplitude (µV) and standard deviations of the − 450 to 0 ms interval of the pre-stimulus ERP components during the three tasks.

	DRT	SRT	Passive
Prefrontal	− 2.02 ± 0.53	− 0.61 ± 0.24	+ 0.02 ± 0.08
Central	− 2.18 ± 0.62	− 1.97 ± 0.48	− 0.36 ± 0.14
Parieto-Occipital	− 1.62 ± 0.48	− 1.81 ± 0.53	− 1.83 ± 0.51

Regarding the TSE, t-tests against zero for evoked activity were only significant (t_(25)_ = 2.91, p < 0.01; Cohen’s d: 0.419) for the subdelta band for all ROIs. T-tests against zero for induced activity were significant (t_(25)_ > 2.74; p < 0.05; Cohen’s d: 0.372) for the delta, theta, alpha, and beta bands (not for subdelta and gamma) for all ROIs. Table 2 presents the mean amplitude of evoked (sub-delta) and induced (delta, theta, alpha, and beta) pre-stimulus TSE in the 450 to 0 ms epoch for the three ROIs.

**Table 2:** Mean amplitude (µV) and standard deviations of the pre-stimulus TSE during the three tasks and the four frequency bands

		DRT	SRT	Passive
**Evoked** Subdelta 0.5–2 Hz	Prefrontal	− 0.71 ± 0.40	− 0.46 ± 0.18	0.28 ± 0.17
	Central	− 0.75 ± 0.15	− 0.69 ± 0.11	− 0.06 ± 0.19
	Parieto-Occipital	− 0.29 ± 0.03	− 0.43 ± 0.10	− 0.40 ± 0.13
Induced Delta 2–4 Hz	Prefrontal	− 0.75 ± 0.14	− 0.34 ± 0.13	− 0.10 ± 0.21
	Central	− 1.08 ± 0.37	− 0.87 ± 0.40	− 0.26 ± 0.16
	Parieto-Occipital	− 0.49 ± 0.27	− 0.61 ± 0.26	− 0.68 ± 0.18
Induced Theta 4–8 Hz	Prefrontal	− 0.28 ± 0.11	− 0.15 ± 0.08	− 0.06 ± 0.05
	Central	− 0.26 ± 0.08	− 0.24 ± 0.03	− 0.05 ± 0.07
	Parieto-Occipital	− 0.25 ± 0.07	− 0.27 ± 0.21	− 0.26 ± 0.09
Induced Alpha 8–13 Hz	Prefrontal	0.14 ± 0.09	0.19 ± 0.06	0.20 ± 0.08
	Central	0.21 ± 0.06	0.29 ± 0.11	0.23 ± 0.03
	Parieto-Occipital	0.28 ± 0.13	0.47 ± 0.17	0.53 ± 0.16
Induced Beta 13–30 Hz	Prefrontal	0.07 ± 0.02	+ 0.05 ± 0.03	+ 0.09 ± 0.02
	Central	0.09 ± 0.05	+ 0.19 ± 0.07	+ 0.13 ± 0.03
	Parieto-Occipital	0.07 ± 0.07	+ 0.15 ± 0.09	+ 0.21 ± 0.11

**Fig. 4:**
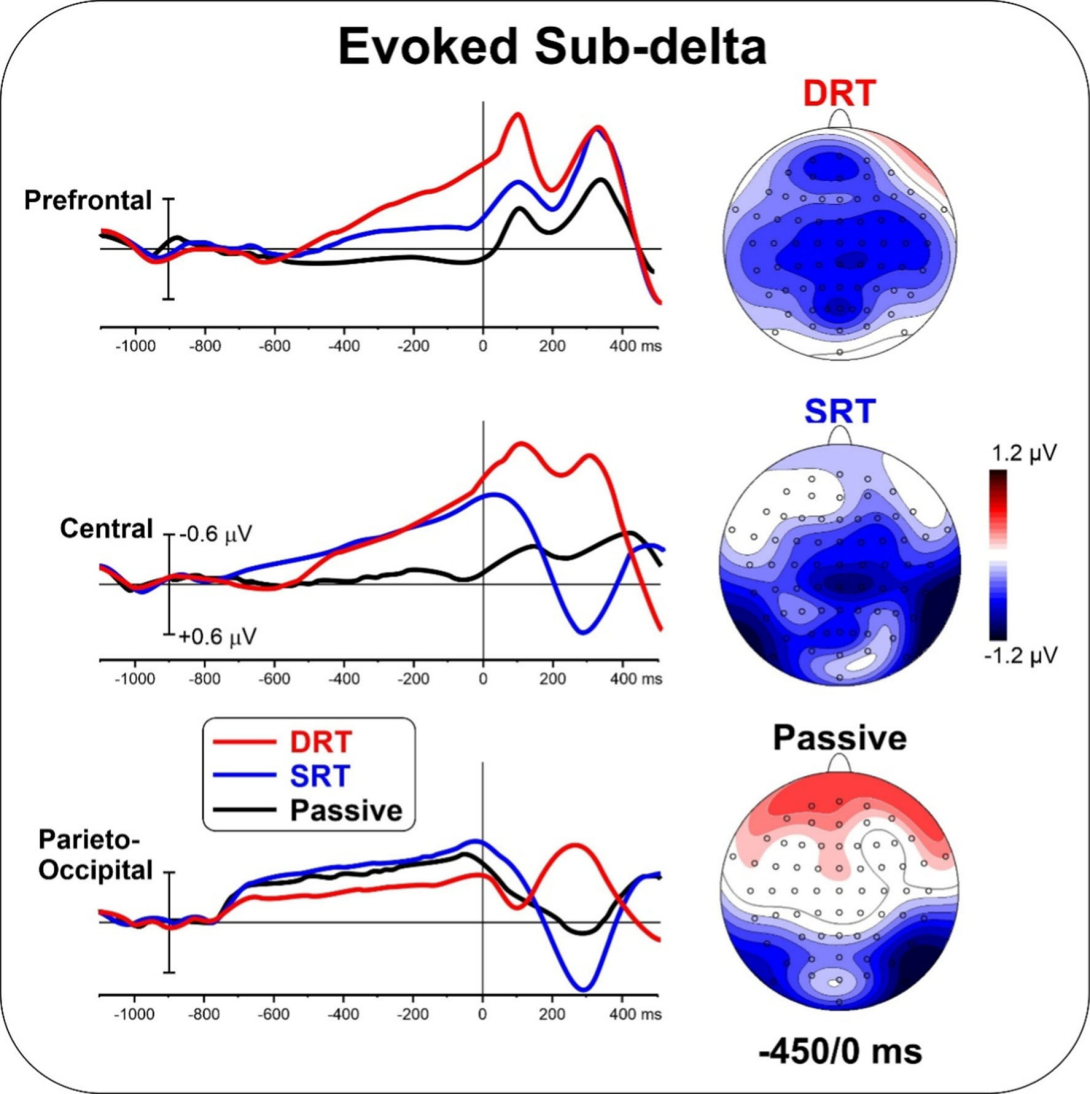
The left panel shows the overlapping evoked TSE waveforms in the subdelta band for the three considered ROIs during the three tasks. The right panel shows the scalp topography (top-flat view) for the three tasks in the − 450/0 ms time window

Figure 4 shows evoked TSE waveforms and topographic mapping in the sub-delta band for the tasks used and ROIs. The DRT showed prominent negative activities in the prefrontal, central and parieto-occipital areas. For the SRT, activity in the central and bilateral parieto-occipital negative foci were present. Bilateral negative activity in the parieto-occipital area and positive activity in a small frontal area were present during the passive task. The evoked sub-delta waveforms resembled the pre-stimulus ERP waveforms and topography in Fig. 3.

ANOVA on the prefrontal area showed a significant effect of Task [F_(2,50)_ = 35.54; p < 0.001, pη^2^ = 0.597]. Post hoc comparisons showed that the activity in the prefrontal area during the DRT was larger than during the SRT (p < 0.001) and passive task (p < 0.001). In addition, the SRT was larger (p < 0.001) than that of the passive task. ANOVA on the central area showed a significant effect of Task [F_(2,50)_ = 14.42; p < 0.001, pη^2^ = 0.375]. Post hoc comparisons showed smaller activity in the central area during the passive task (p < 0.001) than during the DRT and the SRT, which did not differ each other. ANOVA on the parieto-occipital area was not significant [F < 1]. The correlation between ERP and evoked sub-delta TSE was significant for all ROIs and tasks [r = 0.38, F_(1,25)_ = 13.83, p < 0.005 uncorrected, p < 0.01 Bonferroni corrected].

**Fig. 5:**
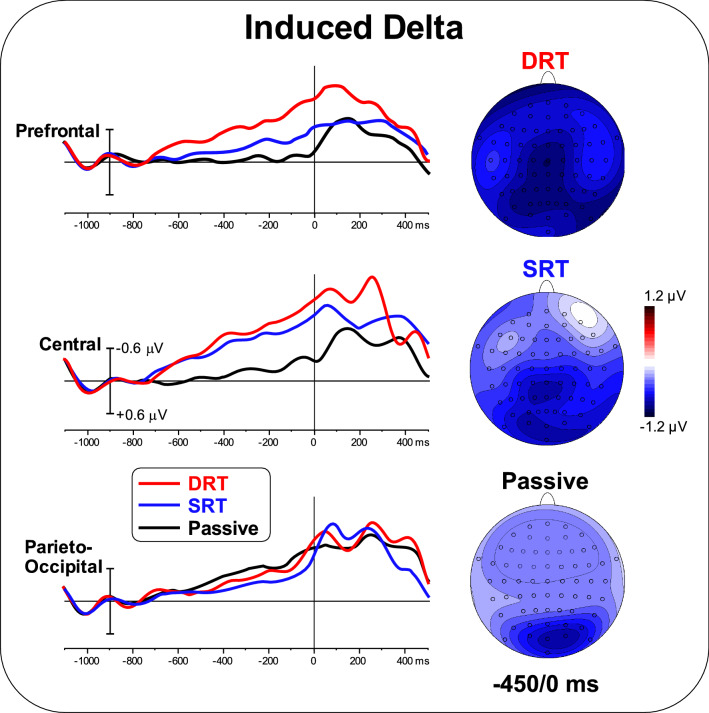
The left panel shows the overlapping TSE waveforms induced in the delta band for the three ROIs during the three tasks. The right panel shows the scalp topography (top-flat view) for the three tasks in the − 450 to 0 ms time window

Figure 5 shows the TSE waveforms induced in the delta band for the included tasks and ROIs. The waveforms show a growing trend with time, analogous to that observed in Fig. 3 for ERPs. Additionally, amplitude modulation according to task follows a similar trend. The topographic mapping of the delta band for the DRT showed negative activity in prefrontal, central and bilateral parieto-occipital areas. For the SRT, negative activity in the central and parieto-occipital ROIs were present. Negative activity in the parieto-occipital ROI was present in the passive task. ANOVA on the prefrontal area showed a significant effect of Task [F_(2,50)_ = 8.21; p < 0.01, pη^2^ = 0.247]. Post hoc comparisons showed that the activity in the prefrontal area during the DRT was larger than during the SRT (p < 0.01) and passive task (p < 0.005). In addition, the SRT was larger (p = 0.05) than the passive SRT. ANOVA on the central area showed a significant effect of Task [F_(2,50)_ = 13.17; p < 0.001, pη^2^ = 0.345]. Post hoc comparisons showed smaller activity in the central area during the passive task (p < 0.01) than during the DRT and the SRT, which did not differ from each other. ANOVA on the parieto-occipital area was not significant [F < 1]. The correlation between ERP and induced delta TSE was significant for the prefrontal and central ROIs during the SRT and DRT [r = 0.31, F_(1,25)_ = 11.02, p < 0.005 uncorrected, p < 0.05 Bonferroni corrected].

**Fig. 6:**
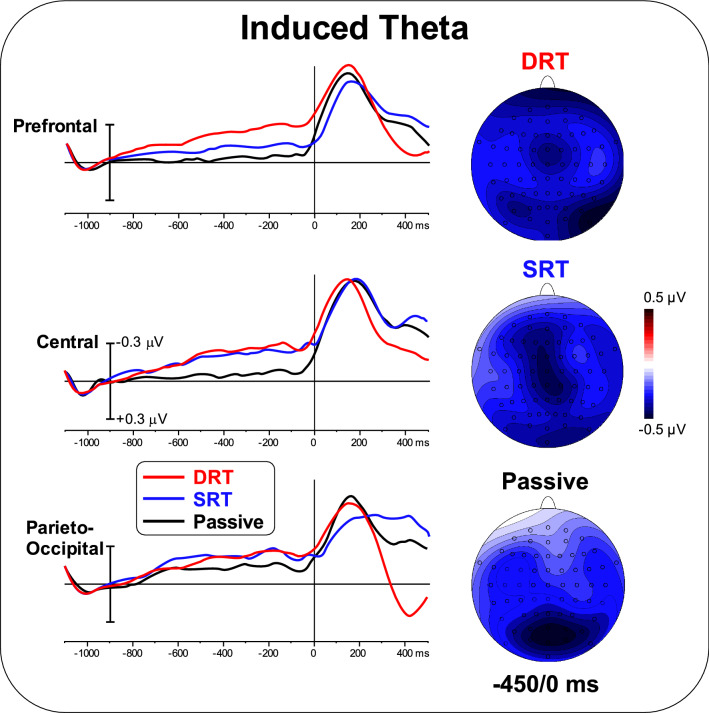
Pre-stimulus TSE waveforms and topographical maps (-450/0 ms) in the theta band. (2-column)

Figure 6 shows the induced TSE waveforms and topographical maps in the theta band. Negative activity in the prefrontal, central and bilateral parieto-occipital ROIs was present in the DRT, while only negative activity in the central and parieto-occipital ROIs were observed in the SRT. In the passive task, activity was observed only over parietal-occipital regions. ANOVA on the prefrontal area showed a significant effect of Task [F_(2,50)_ = 5.73; p < 0.01, pη^2^ = 0.186]. Post hoc comparisons showed that the DRT amplitude was larger than during the SRT (p < 0.05) and the passive task (p < 0.05), which did not differ. ANOVA on the central area showed a significant effect of Task [F_(2,50)_ = 5.29; p < 0.01, pη^2^ = 0.175]. Post hoc comparisons showed smaller activity during the passive task (p < 0.05) than during the DRT and the SRT, which did not differ. ANOVA on the parieto-occipital area was not significant [F < 1]. The correlation between ERP and induced theta TSE was significant for prefrontal and central ROIs during the DRT, for the central ROI during the SRT, and for the parieto-occipital ROI during the passive task [r = 0.28, F_(1,25)_ = 9.78, p < 0.005 uncorrected, p < 0.05 Bonferroni corrected].

**Fig. 7:**
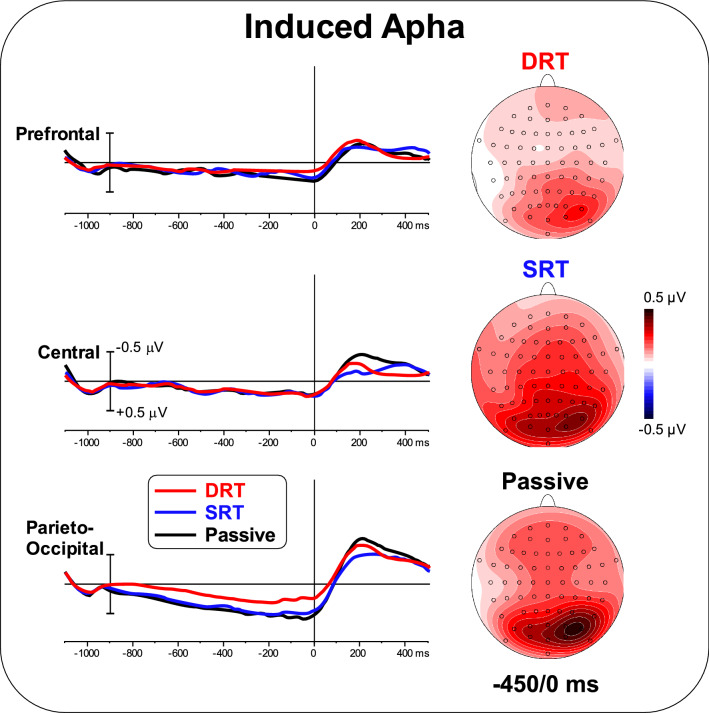
Pre-stimulus TSE waveforms and topographical maps (-450/0 ms) in the alpha band

Figure 7 shows the induced TSE waveforms and topographical maps in the alpha band. Positive activity in the parieto-occipital area was observed in all tasks, with topography slightly extending over central and frontal areas during the SRT and the passive task. ANOVA on the prefrontal and central areas was not significant [F < 1]. ANOVA on the parieto-occipital area showed a significant effect of Task [F_(2,50)_ = 4.71; p < 0.05, pη^2^ = 0.158]. Post hoc comparisons showed smaller activity during the DRT (p < 0.05) than during the SRT and the passive task, which did not differ. The correlation between ERP and induced alpha TSE was significant for the parieto-occipital ROI during the passive task [r=-0.33, F_(1,25)_ = 12.74, p < 0.005 uncorrected, p < 0.05 Bonferroni corrected].

**Fig. 8:**
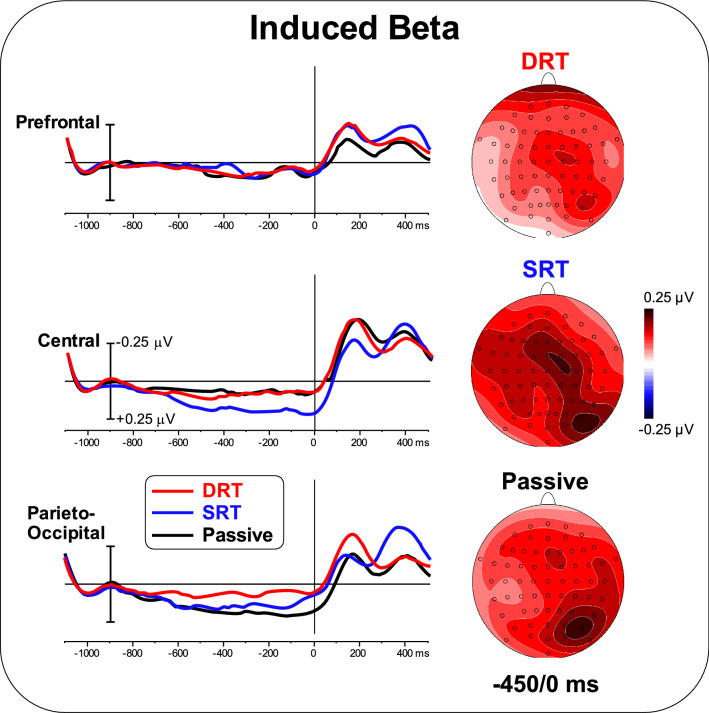
Pre-stimulus TSE waveforms and topographical maps (-450/0 ms) in the beta band

Figure 8 shows the induced TSE waveforms and topographical maps in the beta band. Diffuse positive activity with a small focus of activity in the central and right parieto-occipital areas was observed in the DRT. The topographical distribution of activity during the SRT was similar to that during the DRT, although more marked. In the passive task, a parieto-occipital focus was present. ANOVA on the prefrontal area was not significant [F < 1]. ANOVA on the central area showed a significant effect of Task [F_(2,50)_ = 3.84; p < 0.05, pη^2^ = 0.133]. Post hoc comparisons showed larger activity during the SRT (p < 0.05) than during the DRT and the passive task, which did not differ. ANOVA on the parieto-occipital area showed a significant effect of Task [F_(2,50)<_4.13; p < 0.05, pη^2^ = 0.142]. Post hoc comparisons showed smaller activity during the DRT (p < 0.05) than during the SRT and the passive task, which did not differ. The correlation between ERP and induced beta TSE was significant for the central ROI during the SRT and for the parieto-occipital ROI during the SRT and passive task [r=-0.30, F_(1,25)_ = 9.88, p < 0.005 uncorrected, p < 0.05 Bonferroni corrected].

## Discussion

It is important to highlight that this analysis confirms previous work regarding the first aim of ERPs. The vN is present in all three tasks (passive, SRT and DRT); the BP is present in SRT and DRT; and the pN is present in DRT only (Berchicci et al. 2012; Bianco et al. 2017, 2019a, b; Di Russo et al. 2017; Russo et al. 2019; Mussini et al. 2021). Anticipation of upcoming events has frequently been associated with slow negative cortical potentials, including the CNV (expectancy), BP (motor), pN (cognitive) (Van Boxtel and Böcker 2004) and vN (sensory) (Bianco et al. 2019a, b). In addition, several authors have suggested that slow cortical potentials might be related to the need to act (Schmiedt-Fehr et al. 2010), as is the case of the two tasks (SRT and DRT) considered in the present study.

We characterized the spectral dynamics of anticipatory pre-stimulus ERPs in visuomotor tasks. The results showed that the pN, BP, and vN components were clearly composed of the sub-delta frequency band (0.5-2 Hz) phase-locked with the stimulus onset. No other bands of evoked TSE were present. However, it is known that the slow bands are more influenced by slow drifts in the electrical recording and represent a threat to our study. Nevertheless, the waveform, scalp topography, and modulation of the ERP components as a function of the task were exactly reproduced by the evoked subdelta band. Significant correlations were present for all components in all tasks, confirming this finding.

In addition, we found significantly induced (non-phase) activity in the same time interval as the pre-stimulus ERPs (pN, BP, and vN), suggesting unknown mechanisms for traditional analyses (evoked and phase activity) (Di Russo et al. 2020; Ragazzoni et al. 2019; Sarrias-Arrabal et al. 2021; Vázquez-Marrufo et al. 2020b). For instance, induced delta and induced theta activity showed modulations (desynchronization) within the temporal range of pN and BP components in anterior and central areas but not in parieto-occipital areas. The desynchronization of the induced delta and theta bands suggests the need to decrease these frequencies so that other bands (synchronization) can perform their functions (Sarrias-Arrabal et al. 2021; Vázquez-Marrufo et al. 2020b). Future studies are needed to clarify the functional role of desynchronization of the non-phase delta and theta band during the expectancy interval when vN, BP, and pN ERPs arise (synchronization).

In contrast to delta and theta, the induced alpha band was modulated by task only in the occipital regions. In the present TSE analysis, the induced alpha activity was shown as a positive wave reflecting synchronization of neural activity. This band was confined to parieto-occipital areas as the vN component. This result was also consistent with the report that alpha oscillations are generally evident in posterior and occipital regions (Rodríguez-Hernández et al. 2017).

Based on recent studies about inducing alpha activity (Sarrias-Arrabal et al. 2021; Vázquez-Marrufo et al. 2020), decreased induced alpha activity may reflect a decrease in background activity to improve stimulus processing. If the vN component has been associated with anticipatory sensory information in extrastriatum brain areas (Di Russo et al. 2019), it is reasonable to think that in the DRT task, alpha synchronization prior to the onset of the stimuli is reduced to enhance visual processing to distinguish between target and non-target stimuli.

In line with previous works, synchronization observed in the beta band seems to contribute to the generation of BP. Earlier studies have found an increase in beta activity in Go trials compared to No-go trials (Fründ et al. 2008). The increase in the beta band may represent the synchronization of the local neural population for better performance when a stimulus is displayed. It is possible that this increase in beta represents motor readiness. In the present study, the beta was more evident in central regions (BP). This ERP has been related to motor readiness (Kornhuber and Deecke 1965; Shibasaki and Hallett 2006). According to this view, the larger beta activity in the SRT respective to the passive and DRT make sense. SRT is the only task that requires emitting a motor response in every trial, whereas, in the case of the passive task, participants do not have to prepare a response, and in DRT, there is more than one response. In DRT, subjects did not know which visual stimuli would appear (target or not target), and excessive motor readiness for any upcoming sensory stimulus could increase the false alarm rate.

This study was limited to the analysis of the TSE amplitudes; future investigation should also focus on the phase relations of the TSE.

## Conclusion

ERPs, which have been previously described in cueing paradigms (BP, pN, and vN), were observed in this paradigm without cues, indicating that preparatory processes are present during the execution of cognitive tasks. BP, pN, and vN were related to motor preparation, inhibition, and sensory preparation, respectively. Moreover, the spectral counterpart of these modulations was mainly concentrated in the sub-delta band (0.5-2 Hz).

Induced activity has been reported during the interval where ERPs occurred, suggesting that other cognitive processes are modulated to improve preparation for incoming stimuli. The changes in this activity were reflected in desynchronizations (delta and theta) and synchronizations (alpha and beta) with diverse cognitive functions.

## Data Availability

Data and materials will be made available upon request.

## Code Availability

Codes will be made available upon request.
